# Primary small cell carcinoma of the esophagus: clinicopathological study of 44 cases

**DOI:** 10.1186/1471-2407-14-222

**Published:** 2014-03-25

**Authors:** Wei-Wei Chen, Feng Wang, Dong-Sheng Zhang, Hui-Yan Luo, Zhi-Qiang Wang, Feng-Hua Wang, Miao-Zhen Qiu, Chao Ren, Xiao-Li Wei, Wen-Jing Wu, Yu-Hong Li, Rui-Hua Xu

**Affiliations:** 1 Department of Medical Oncology and State Key Laboratory of Oncology in South China, Sun Yat-sen University Cancer Center, Guangzhou, China; 2 Department of Medical Oncology, Sun Yat-sen University Cancer Center, 651 Dong Feng Road East, Guangzhou 510060, China

**Keywords:** Small cell carcinoma, Esophagus, Prognosis, Lgr5

## Abstract

**Background:**

Primary small cell carcinoma of the esophagus (SCCE) is a highly aggressive disease characterized by early dissemination and poor prognosis. Because of the rarity of this disease, few previous studies have investigated the biomarkers associated with its prognosis. Leucine-rich repeat-containing G-protein coupled receptor 5 (Lgr5) is a stem cell marker and a member of the canonical Wnt-signaling cascade. However, the clinical role of Lgr5 in SCCE remains unknown.

**Methods:**

Tissue sections were obtained from 44 patients diagnosed with SCCE and expression of Lgr5 was examined by immunohistochemistry. The correlations between Lgr5 expression, and clinical parameters and prognostic significance were evaluated.

**Results:**

Lgr5 was expressed in SCCE cancer tissues. High Lgr5 expression was significantly correlated with lymph node metastasis (p = 0.003), late stage (p = 0.003) and unfavorable response to chemotherapy (p = 0.013) according to RECIST 1.0 criteria. Patients with higher Lgr5 expression levels had shorter overall survival times than those with lower expression levels.

**Conclusions:**

These results demonstrated that overexpression of Lgr5 was significantly correlated with lymph node metastasis, tumor stage, and response to chemotherapy. Furthermore, high levels of Lgr5 expression appeared to be associated with poorer survival in patients with SCCE.

## Background

Esophageal carcinoma is one of the most common malignancies in China, but primary small cell carcinoma of the esophagus (SCCE) is a relatively rare histopathological type, accounting for only 1-2.8% of esophageal carcinomas [1,2]. SCCE is highly aggressive, and characterized by early dissemination and poor prognosis [3-10]. A total of 5,379 cases of esophageal cancer and 2,061 cases of small cell carcinoma were recorded at Sun Yat-sen University Cancer Center from 1994 to 2012, including 93 cases of SCCE. Little is known about the clinicopathological features of SCCE, and it is necessary to identify biomarkers for predicting prognosis and for distinguishing individuals with unfavorable prognoses.

The Wnt signaling pathway is important for adult tissue maintenance. Perturbations in Wnt signaling cause human cancers [11]. Leucine-rich repeat-containing G-protein coupled receptor 5 (Lgr5) is an orphan G-protein coupled receptor (GPCR) and a stem cell marker first described as a Wnt target gene [12]. It is also a member of the canonical Wnt-signaling cascade, which forms a signaling gradient in the intestinal crypt, thereby regulating cell proliferation and differentiation [13]. Lgr5 was also identified as a marker of poor prognosis in colon, ovary and liver cancers [14,15], and was considered to be involved in tumorigenesis in Barrett’s esophagus and esophageal adenocarcinoma [16].

However, the expression and potential clinical significance of Lgr5 in SCCE has not been determined. In this study, we analyzed the expression levels of Lgr5 and their relationships with clinicopathological features in 44 patients with SCCE.

## Methods

### Tumor tissues and patient information

A total of 44 paraffin-embedded samples were obtained from patients diagnosed with SCCE from January 1 1994 to January 1 2012 at Sun Yat-sen University Cancer Center. The diagnosis of SCCE had been confirmed by the Pathology Department of Sun Yat-sen University Cancer Center, based on the 2000 World Health Organization histological criteria for esophageal small cell carcinoma [17] and 2004 histological criteria for pulmonary small cell carcinoma [18]. As described previously [10], tumor cells were characterized by small, spindle-like, round or ovoid shape, scarce cytoplasm, indistinct cell borders, and an inconspicuous or absent nucleolus. Information on the neuroendocrine markers neuron-specific enolase (NSE), synaptophysin (Syn), chromogranin A (CgA) and CD56 were obtained from patient pathology reports. All patients were positive for CgA and/or Syn expression; about 70.5% of patients were Syn-positive, 84.1% were CgA-positive, 56.8% of cases were NSE-positive and 34.1% were CD56-positive.

Informed consent was obtained from all patients and the study was approved by the Research Ethics Committee of Sun Yat-Sen University. No patients had received any treatment prior to surgery or biopsy. Of the 44 samples, 29 were obtained at surgery and 15 by biopsy. The clinicopathological records of all patients were reviewed. The American Joint Committee on Cancer (AJCC) clinical and pathologic staging system was adopted for all patients. Overall survival (OS) was defined as the time from the date of diagnosis to the point of death or the last follow-up.

### Immunohistochemistry (IHC)

IHC was performed to study the expression of Lgr5 in SCCE tissues, as described previously [19]. Briefly, paraffin-embedded tissue sections were baked at 60°C for 2 h, deparaffinized, and rehydrated. After treatment with 3% hydrogen peroxide for 30 min, the sections were put in a high-pressure environment for antigen retrieval. Tissue sections were incubated with rabbit anti-Lgr5 (1:400; Abcam, United states) overnight at 4°C, then treated with anti-rabbit secondary antibody for 40 min, followed by treatment with diaminobenzidine tetrahydrochloride (DAB), and counterstaining with hematoxylin. Human colon cancer tissues with strong Lgr5 staining were used as positive controls, based on previous reports [20]. Lgr5 immunostaining was evaluated by two independent observers who were blinded to the clinicopathological characteristics of the patients. Immunostaining scores were awarded by two independent observers according to the percentage and intensity of the stained cells. Positivity values were as follows: 0 (<10%), 1 (10–25%), 2 (25–50%), 3 (50–75%), and 4 (>75%). Intensity values were as follows: 0 (negative), 1 (weak staining), 2 (moderate staining), and 3 (strong staining). The final score was calculated by multiplying the above two values. For subsequent analysis, high expression was defined as a final score >4 and low expression was a score ≤4.

### Statistical analysis

Statistical analysis was carried out using SPSS 16.0. The significance of correlations between biomarker expression levels and clinical features were calculated using *χ*^2^ tests. Survival curves were displayed by Kaplan–Meier analysis and differences in survival were assessed by log-rank tests. A two-sided α-error of less than 5% (p < 0.05) was considered as statistically significant.

## Results

### Expression of Lgr5 in SCCE and correlation between expression and clinicopathologic features

**Figure 1:**
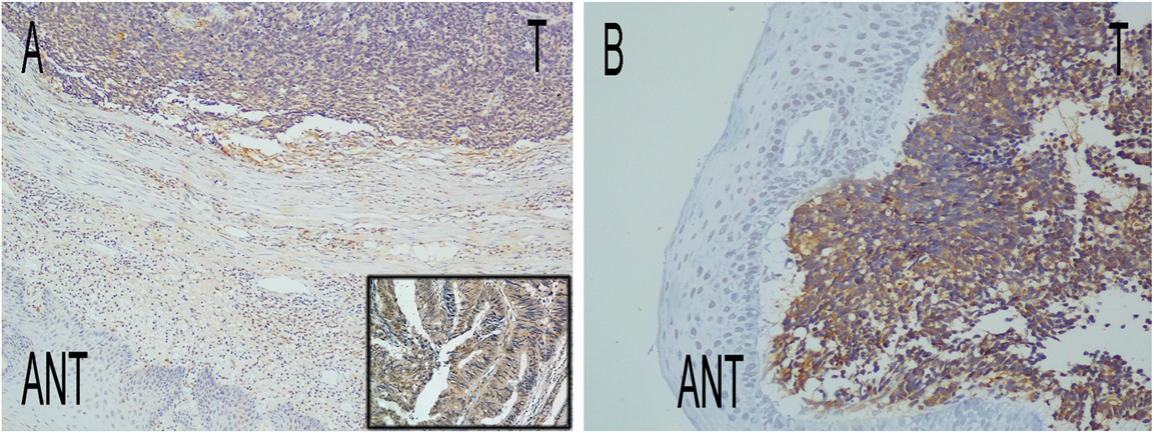
**Immunohistochemical staining of Lgr5 in cancerous tissue and adjacent normal mucosa. A**: Higher levels of Lgr5 expression were observed in tumor tissues compared with ANT (×100 magnification). Colon cancer tissue was used as a positive control (insert). **B**: Higher levels of Lgr5 expression were observed in tumor tissues compared with ANT (×400 magnification). Abbreviations: T: tumor, ANT: adjacent normal tissue.

**Figure 2:**
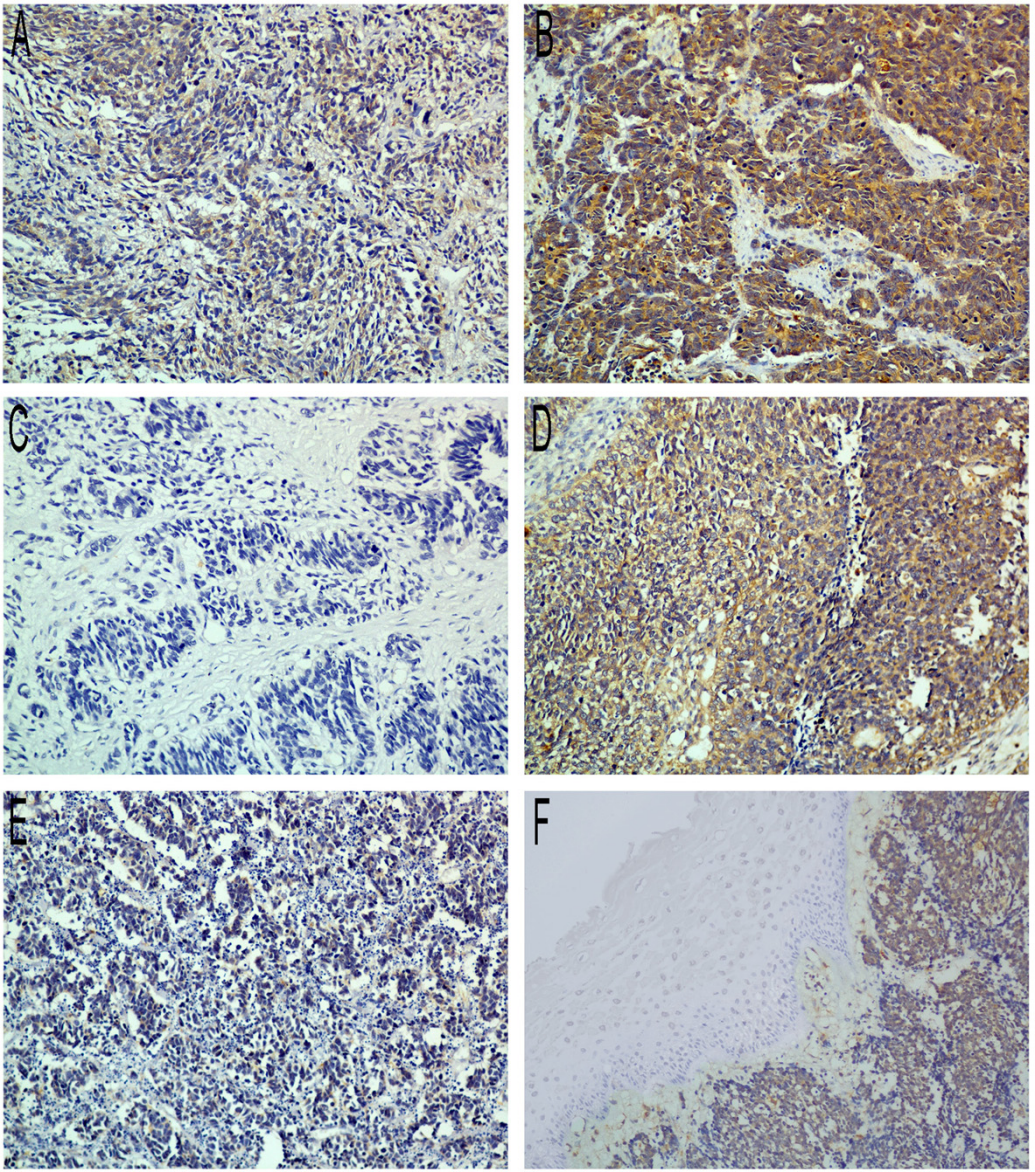
**Immunohistochemical staining of Lgr5 in small cell carcinoma of the esophagus (×400 magnification). A**: Lgr5 expression in stage I patients (representative slide). **B**: Lgr5 expression in stage IV patients (representative slide). **C**: Lgr5 expression in patients without lymph node metastasis (representative slide). **D**: Lgr5 expression in patients with lymph node metastasis (representative slide). **E**: Lgr5 expression in patients who achieved complete response during chemotherapy (representative slide). **F**: Lgr5 expression in patients evaluated with progressive disease during chemotherapy (representative slide).

Detailed information was collected for 44 patients (Table 1). The median follow-up time for all patients was 11.1 months (3–84.9 months). Tissue sections were subjected to IHC to investigate Lgr5 expression levels and to correlate these with clinicopathological features. Lgr5 was localized mainly in the cytoplasm of cancer cells (Figure 1). Adjacent normal esophageal tissue (ANT) was available from five patients, two of whom showed higher Lgr5 expression levels in tumor tissues than in ANT (representative slide shown in Figure 1). According to the IHC scoring system, 47.7% of tumors showed high cytoplasmic expression of Lgr5. High Lgr5 expression levels were significantly correlated with lymph node metastasis (p = 0.003), late stage (p = 0.003) and unfavorable response to chemotherapy (p = 0.013) according to RECIST 1.0 criteria (Table 2, representative slides shown in Figure 2). However, Lgr5 expression was not correlated with sex, age, tumor type, tumor location, tumor length or distant metastases.

**Table 1:** Clinicopathological characteristics and Lgr5 expression in tumors from 44 patients with SCCE

**Variable**	**No.of patients**	**%**
Gender		
Male	33	75.0
Female	11	25.0
Age		
>60	15	34.1
<=60	29	65.9
PS score		
>1	4	9.1
<=1	40	90.9
Tumor length(39 available)		
> = 5 cm	21	53.8
<5 cm	18	46.2
Tumor location		
Upper thoracic segment	7	15.9
Middle thoracic segment	22	50.0
Lower thoracic segment	15	34.1
AJCC stage(43 available)		
I/II	19	44.2
III/IV T classification(40 available)	24	55.8
T < =2	20	45.5
T > 2	20	45.5
N classification(42 available)		
N0	16	36.4
N+	26	59.1
M classification(43 available)		
M0	33	75.0
M1	10	22.7
Treatment approach		
Surgery		
No	11	25.0
Yes	33	75.0
Chemotherapy		
No	16	36.4
Yes	28	63.6
Radiotherapy		
No	16	36.4
Yes	28	63.6
Lgr5 expression		
Low	23	52.3
High	21	47.7

**Table 2:** Correlation between clinicopathological characteristics and Lgr5 expression in patients with SCCE

	**Lgr5 expression**		
**Characteristics**	**Low or none No. cases**	**High No. cases**	**Chi-square test P-value**
Gender			.601
male	18	15	
female	5	6	
Age			.241
>60	6	9	
<=60	17	12	
Tumor length(39 available)			.256
> = 5 cm	9	12	
<5 cm	11	7	
Tumor location			.721
Upper thoracic segment	3	4	
Middle thoracic segment	11	11	
Lower thoracic segment	9	6	
AJCC stage(43 available)			.003
I/II	15	4	
III/IV	8	16	
T classification(40 available)			.206
T < =2	12	8	
T > 2	8	12	
N classification(42 available)			.003
N0	13	3	
N+	9	17	
M classification(43 available)			.329
M0	19	14	
M1	4	6	
Chemotherapy response(19 available)			.013
Reached CR/PR	5	1	
Did not reach CR/PR	3	10	

### Association between Lgr5 expression and patient survival

**Figure 3:**
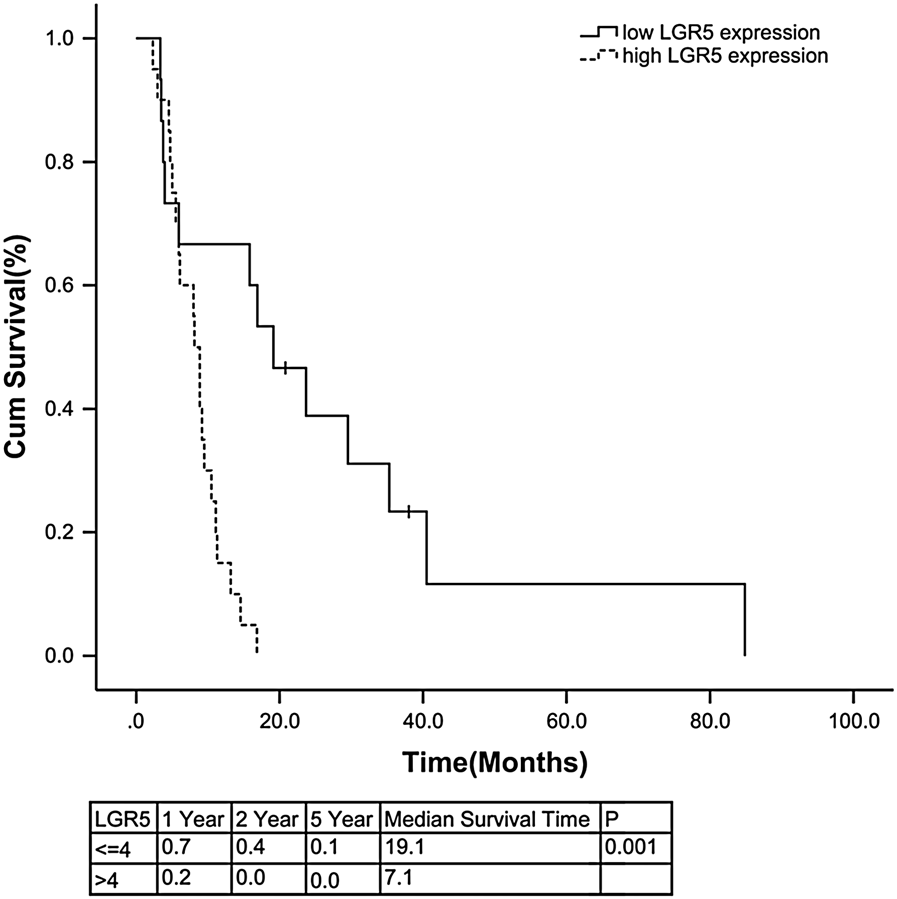
**Kaplan–Meier curves for lower versus higher expression levels of Lgr5.** Nine patients were lost to follow-up. Overall survival time was available for 35 patients.

High expression of Lgr5 was associated with a shorter overall survival time (Figure 3, p = 0.001). The overall 1-, 2-, and 5-year cumulative survival rates in patients with high expression levels of Lgr5 were 20%, 0%, and 0%, respectively, compared with 70%, 40%, and 10% in patients with low Lgr5 expression. Cox regression analysis identified tumor length (p = 0.043), lymph node involvement (p = 0.04) and chemotherapy (p = 0.006) as independent prognostic factors (Table 3).

**Table 3:** Univariate and multivariate analyses of prognostic parameters in patients with SCCE

	**Univariate analysis**		**Multivariate analysis**			
**Variable**	**No.**	**P**	**HR**	**95.0% CI**		**p**
Tumor length		.045	.288	.086	.963	.043
> = 5 cm	21					
<5 cm	18					
Lgr5 expression		.001	1.519	.346	6.659	.580
Low	23					
High	21					
T classification		.725	.760	.276	2.090	.595
T < =2	20					
T > 2	20					
N classification		.170	3.726	1.063	13.061	.040
N0	16					
N+	26					
M classification		.096	4.861	.902	26.212	.066
M0	33					
M1	10					
Chemotherapy		.088	.229	.081	.649	.006
No	16					
Yes	28					
Surgery		.207	1.222	.257	5.822	.801
No	11					
Yes	33					

## Discussion

SCCE is a highly metastatic disease with a median survival time of less than 1 year [21]. The recurrence rate after surgery is very high, even in patients with early-stage disease [22]. However, the aggressive features and poor prognosis of SCCE found in clinical practice have not yet been linked to any specific biomarkers. Recent genetic discoveries based on tumor genome sequencing suggest that the Wnt pathway plays important roles in tumor biology [23]. Activation of the Wnt pathway releases β-catenin, which interacts with T cell factor family members to activate the transcription of downstream target genes [24]. R-spondins potently enhance β-catenin signaling and have been implicated in human disease and malignancy [25,26]. Lgr5 is a receptor for R-spondin and was shown to activate β-catenin signaling when bound to R-spondins [25,27,28], and may be associated with tumorigenesis via the Wnt pathway.

Iuga et al. [29] reported that Lgr5 was a novel IHC marker for gastrointestinal neuroendocrine tumors; 88% of primary neuroendocrine tumors and 87% of metastases stained positive for cytoplasmic Lgr5. Moreover, Lgr5 stained positive in most cases expressing CgA and Syn (34/38). These findings were consistent with our study, which also found high Lgr5 expression levels in SCCEs, which were rich in CgA and Syn. von Rahden et al. [16] reported LgR5 expression in 35 of 41 (85%) patients with esophageal adenocarcinomas with Barrett’s esophagus, and in 16 of 19 (81%) without Barrett’s esophagus. Becker et al. [30] also detected Lgr5 expression in early esophageal squamous cells. Based on the above findings, we examined Lgr5 expression in SCCE.

Of the 44 patients with SCCE examined by IHC, only 15.8% who presented with high levels of Lgr5 survived for longer than 1 year after diagnosis (data not shown). The median survival in patients with high expression levels of Lgr5 was 7 months, which was 11 months shorter than in patients with lower expression of Lgr5. Less than 20% of patients (18 patients) had survived longer than 2 years at the end of follow-up, none of whom presented with high Lgr5 expression. Our results indicated that high levels of Lgr5 expression were significantly correlated with lymph node metastasis and late stage (stage III/IV), suggesting that high expression of Lgr5 might predict a poor prognosis. Indeed 62% of patients with lymph node metastases failed to survive for longer than 1 year, in accordance with a previous study in which esophageal cancer patients with more than four involved lymph nodes showed similar survival to patients with M1 disease [31]. In Cox regression analysis, lymph node involvement was an independent prognostic factor, which might explain the association between high expression of Lgr5 and poor prognosis.

SCCE is a systemic disease [21]. Chemotherapy provides the backbone of SCCE therapy [21,32], which was in accordance with the fact that chemotherapy was an independent prognostic factor in the present study. Lgr5 expression may also predict response to chemotherapy in SCCE. High Lgr5 expression was significantly correlated with unfavorable response in this study; only 33% of patients with high expression achieved partial/complete responses during chemotherapy. Additionally, Lgr5 may also predict chemotherapy response in colorectal cancer [33].

A recent study suggested that GPCRs and their signal transduction pathways may provide promising new therapeutic approaches [34]. They are involved in the control of blood pressure, maintenance of kidney function, occurrence of neurological diseases and the progression of cancer [34]. Approximately 36% of currently-marketed drugs target GPCRs [35]. Lgr5 has been suggested to be involved in cancer progression through regulation of the Wnt signaling pathway [25,27,28]. Lgr5 knockdown was shown to induce cell death [36], and furthermore, a recently-developed monoclonal antibody (KM4056) was reported to have potent complement-dependent cytotoxicity activity in vitro, and to show strong anti-tumor activity in vivo [37]*.* Lgr5 thus remains a potential for targeted therapy.

Overall, the results of this study suggest that Lgr5 protein expression may represent a possible prognostic marker in SCCE patients. However, these results need to be validated by further studies with larger sample sizes and in randomized patient cohorts before Lgr5 IHC can be used in clinical applications.

## Conclusions

In summary, overexpression of Lgr5 was significantly correlated with lymph node metastasis, tumor stage and response to chemotherapy, while high levels of Lgr5 expression were also associated with poor survival in patients with SCCE.

## Abbreviations

SCCE: Primary small cell carcinoma of esophagus; GPCR: Orphan G-protein coupled receptor; AJCC: American Joint Committee on Cancer; OS: Overall survival; ANT: Adjacent normal tissue.

## Competing interests

The authors declare that they have no competing interests.

## Authors’ contributions

WC, FW carried out the immunohistochemistry staining and drafted the manuscript. WC, FW, DZ, HL, ZW^,^ FW, MQ, CR, XW and WW participated in the design of the study and performed the statistical analysis. WC, FW, YL and RX conceived of the study, and participated in its design and coordination and helped to draft the manuscript. All authors read and approved the final manuscript.

## Pre-publication history

The pre-publication history for this paper can be accessed here:

http://www.biomedcentral.com/1471-2407/14/222/prepub
